# The inverted syringe technique for management of inverted nipples in breastfeeding women: a pilot randomized controlled trial

**DOI:** 10.1186/s13006-022-00452-1

**Published:** 2022-02-05

**Authors:** Mona Nabulsi, Rayan Ghanem, Hanan Smaili, Ali Khalil

**Affiliations:** 1 Department of Pediatrics and Adolescent Medicine, Faculty of Medicine, American University of Beirut, Beirut, Lebanon; 2 Present Address: Department of Obstetrics and Gynecology, Faculty of Medicine, American University of Beirut, Beirut, Lebanon

**Keywords:** Inverted nipple, Breastfeeding, Inverted syringe technique

## Abstract

**Background:**

Women with inverted nipples may struggle with breastfeeding and may stop exclusive breastfeeding before six months. The use of an inverted syringe to evert the nipples was successful in achieving high rates of infant latching and exclusive breastfeeding in case series but has not been tested in clinical trials. This open label, parallel group, randomized clinical trial investigated whether the use of the inverted syringe technique in women with inverted nipples would increase exclusive breastfeeding rate at one month, as compared to standard care.

**Methods/Design:**

Between June 2018 and January 2020, healthy pregnant women (*N*=54) with grades 1 or 2 inverted nipples were randomly allocated to standard care or to an experimental group that used the inverted syringe technique to evert the inverted nipple prior to every breastfeeding. The primary outcome measure was the rate of exclusive breastfeeding at one month. Secondary outcomes included the rates of exclusive breastfeeding at three and six months, any breastfeeding at one, three, and six months, nipple eversion, successful infant latching, breastfeeding-associated complications, maternal satisfaction with breastfeeding, maternal quality of life, and adverse events. Descriptive and bivariate analyses were conducted according to the intention to treat principle.

**Results:**

Participants in the experimental group were less likely to be exclusively breastfeeding at one (*RR* = 0.65, 95% *CI*: 0.44, 0.95; *n* = 47), and at three months (*RR* = 0.66, 95% *CI*: 0.47, 0.91; *n* = 45), or to practice any breastfeeding at six months (*RR* = 0.54, 95% *CI*: 0.34, 0.87; *n* = 44). Only 14.3% of women in the experimental group complied with the use of the inverted syringe during the first month. Breast pump and breastfeeding-associated complications were more commonly reported in the control group (*p* < 0.05 for both). Both groups had similar rates of nipple eversion, successful infant latching, and similar satisfaction with breastfeeding and quality of life issues.

**Conclusion:**

The inverted syringe technique was not associated with improvement in breastfeeding outcomes of women with inverted nipples. Larger clinical trials are needed to confirm our findings.

**Trial registration:**

ClinicalTrials.gov NCT03529630; Registered May 8, 2018.

**Supplementary Information:**

The online version contains supplementary material available at 10.1186/s13006-022-00452-1.

## Background

The inverted nipple is an abnormality that is present in 3% of females, with bilateral involvement in 86.8% of affected women [1]. A higher prevalence rate of 9.8% has been reported in pregnant women [2]. Nipple inversion is mostly congenital but can be acquired secondary to inflammation such as in mastitis, or due to other conditions such as cancer or breast surgery. Han and Hong classified the severity of nipple inversion into three grades based on the ability to manually pull out the nipple and maintain its projection, and the extent of fibrosis beneath it. Grade 1 inverted nipple has minimal fibrosis, is easily pulled out manually with maintenance of good projection. Grade 2 inverted nipple has moderate fibrosis beneath it, can be pulled out manually but fails to maintain projection. Grade 3 has severe fibrosis and inversion and hence cannot be pulled out manually [3].

Women with inverted nipples often struggle with breastfeeding because of inadequate infant latching that may lead to insufficient milk extraction, maternal frustration, and poor infant satiety, ultimately ending with premature termination of breastfeeding [4–7]. Early weaning from breastfeeding deprives the infant from his mother’s milk which is the ideal nutrition. It may also reduce his chances of other health benefits such as improved growth and development, reduced infections, less risk of chronic diseases, better cognition, and higher intelligence quotient [8–12]. Mothers who discontinue breastfeeding or do not breastfeed may also be at higher risks for certain cancers, chronic diseases such as obesity, diabetes, and depression [8, 10, 13, 14]. The World Health Organization recommends exclusive breastfeeding for the first six months and continuation of breastfeeding with complementary foods until the infant is two years of age [15]. Hence, it is important to provide women with inverted nipples with treatment options that will help them maintain breastfeeding and meet breastfeeding guidelines [15].

Treatment options for inverted nipples include surgical and non-surgical interventions. Historically, corrective surgery was reserved for the severely invaginated nipples (grade 3) that are not amenable to manual extraction, whereas non-surgical methods were indicated for less severe inversion (grades 1 or 2). Non-surgical interventions are several with variable rates of breastfeeding success and/or correction of nipple inversion [16–20]. Hoffman exercises and Woolwich breast shields for example were investigated in a clinical trial by Alexander, et al., and were found to have no effect on nipple anatomy or breastfeeding rates [16]. The Niplette™ (Philips Avent, Andover, MA), which was described in a case series of 22 women by McGeorge [17] applies gentle negative suction over the nipple throughout the day and/or night to extract it, with a reported success rate of 80% for nipple eversion and 100% for breastfeeding. Chakrabarti and Basu [18] applied a rubber band at the nipple base of 19 women with flat or inverted nipples during breastfeeding. Successful latching was present in 60% by the third day, and all women were breastfeeding by 28 days. Kesaree, et al. [19] used an inverted syringe to apply negative pressure around the nipple in eight women. Seven infants were latching by the first week and six were exclusively breastfeeding at six weeks. Although these non-surgical interventions are simple and inexpensive, they may be complicated by adverse events such as nipple infection, bleeding of the nipple or slipping of the rubber band into the infant’s mouth. Except for the study of Alexander, et al., the previous reports were all case series and hence provide low quality evidence on the effectiveness of non-surgical interventions in improving breastfeeding rates in women with nipple inversion. Moreover, these studies had very small sample sizes and lacked power to draw conclusions.

In the present study we aimed to address the existing knowledge gap by conducting the first randomized controlled clinical trial to test whether the use of the inverted syringe technique in healthy term pregnant women with inverted nipples would improve breastfeeding rates.

## Methods

### Study design

This is an open-label, parallel arm, single-center, randomized clinical trial that is reported in accordance with the CONSORT 2010 statement guidelines for reporting parallel group randomized trials [21]. Its protocol was registered in ClinicalTrials.gov (NCT03529630) and published in *Trials* [22]. The study was approved by the Institutional Review Board of the American University of Beirut (Protocol PED.MN.15). Written informed consent was obtained from all participants.

### Participants

Healthy pregnant women presenting to the Women’s Health Center and the Delivery Suite of the American University of Beirut Medical Center, Beirut, Lebanon were approached for enrolment in the study. Inclusion criteria were age at or above 18 years, completed at least 37 weeks of gestation, with one or two flat or inverted nipples of grades 1 or 2 according to Han and Hong’s classification of inverted nipples [3], intending to breastfeed after delivery, and residing in Lebanon for six months after delivery. Exclusion criteria were women with grade 3 inverted nipples, breast conditions that may affect the breast anatomy such as previous breast surgery, not intending to breastfeed, any maternal or infant condition that may interfere with breastfeeding such as critical condition, congenital malformations like cleft lip and/or palate or esophageal atresia, and high-risk pregnancies. Women with twin gestation were not excluded if delivered at term. A trained research assistant verified inclusion criteria, explained the study’s objective and procedures, and obtained written informed consent from all participants.

### Randomization and concealment

Participants were randomly allocated to the experimental or to the control arm in a 1:1 ratio. The random sequence was computer-generated by an independent statistician and concealed by using sequentially numbered opaque sealed envelopes. A participant’s allocation was revealed only after the research assistant obtained her written informed consent.

### Interventions

The trial was open label in view of the nature of the intervention. Participants in the experimental group were trained on the use of the inverted syringe on their first postpartum day in the privacy of their rooms by a trained research assistant. They were instructed to use a 10-cc inverted syringe before each breastfeeding. The mother was shown how to position the base of the inverted syringe over the inverted nipple, and gently pull until the nipple was everted, maintaining it for one minute, after which the syringe was removed, and breastfeeding started. The choice of the suction duration and syringe size were based on the initial description of the intervention by Kesaree, et al. [19]. Mothers were instructed that they may stop using the syringe if the nipple everted spontaneously and the infant was able to latch properly. The participants were also shown how to modify a 10-cc syringe into an inverted one in accordance with the report by Kesaree, et al. [19].

Participants in the control group received standard advice on their infant nutrition, and on treatment of their inverted nipples by their primary physicians. Standard advice on infant nutrition could include exclusive or partial breastfeeding either by direct latching on the mother’s breast, or by using a nipple shield, Niplette™, inverted syringe, expressed maternal milk using a pump, or the use of artificial milk instead of breastfeeding.

### Outcome measures

Our primary outcome measure was the rate of exclusive breastfeeding at one month postpartum. Exclusive breastfeeding was defined as giving the infant maternal milk only, with no other food or drink including water, but allowing oral rehydrating solutions, vitamins, minerals, or other medicines when needed [15]. We considered an infant to be exclusively breastfeeding whether breast milk was provided through direct latching on the mother’s breast or was expressed and offered by cup or bottle. Participants were instructed to continue using the syringe until discontinuation of breastfeeding or complete eversion of the nipple, whichever happened first.

Secondary outcome measures were the rates of exclusive breastfeeding at three and six months; mixed feeding at one, three and six months; nipple eversion (assessed by the research assistant’s examination of the nipple at one month); successful latching on mother’s breast at one month; and breastfeeding complications at one week, and at one, three and six months. Breastfeeding-associated complications were defined as the occurrence of sore nipple, mastitis, breast pain, bleeding, or engorgement in at least one breast. Moreover, we assessed maternal satisfaction with breastfeeding at one week using the validated Arabic Maternal Breastfeeding Evaluation Scale [23]. This scale measures maternal perceived overall quality of her breastfeeding experience. It has 26 items divided into three subscales: Infant Satisfaction/Growth, Maternal Enjoyment/Role Attainment, and Lifestyle/Body. Item responses are scored using a 5-point Likert-type scale ranging from 1 (*strong disagreement*) to 5 (*strong agreement*). The sum of all item scores results in a minimum of 26, and a maximum of 130 points, with higher score indicating higher maternal satisfaction with the breastfeeding experience. We also assessed maternal quality of life at one month using the validated Postpartum Quality of Life Questionnaire [24]. This instrument is a validated tool that measures the quality of life of mothers during the early postpartum period. It is composed of two parts with identical 39 items: satisfaction and importance, and has five domains: psychological/baby, socioeconomic, relational/spouse-partner, relational/family-friends, and health and functioning. Items are scored according to a Likert-type scale that ranges from 1 (very dissatisfied) to 6 (very satisfied). Scores are calculated by weighting each satisfaction response with its paired importance with higher scores reflecting better quality of life.

### Study procedures

At baseline, the research assistant collected data on maternal age, parity, highest educational attainment, employment, monthly household income, previous breastfeeding (Yes/No), grading of the inverted nipple(s), and the longest duration of previous breastfeeding in multiparous women. This was defined as the longest period of previous exclusive breastfeeding (in months) during which a participant breastfed a daughter/son. Moreover, on the first day postpartum, data were collected on the mode of delivery, gestational age, infant’s gender, birth weight, APGAR score, newborn feeding (exclusive breastfeeding/artificial milk/mixed), sore nipple (Yes/No), use of devices to evert the nipple, and compliance with the use of the syringe (experimental group only).

All participants were provided with a diary to document on daily basis in the first month information on the type of infant nutrition (breast milk/formula), number of artificial milk feedings, direct latching on the breast, use of any artificial device to correct the inverted nipple such as nipple shield, Niplette™ or syringe, as well as breastfeeding-associated complications. On the third and seventh day postpartum, reminders about documentation in the diary were sent. In addition, participants in the experimental group were contacted on weekly basis to reinforce the use of the inverted syringe before each breastfeeding. The diaries were collected at one month (+ 10 days) by the research assistant who reassessed the nipple eversion status, recorded the infant’s weight, and administered the Postpartum Quality of Life Questionnaire. At three and six months, information on the infant’s weight and nutrition, as well as breastfeeding-associated complications was collected by telephone.

### Sample size

The sample size was calculated to detect a difference of 35% in the rate of exclusive breastfeeding at one month between the experimental and control groups, with 90% power, and 5% type I error. We hypothesized that 40% of the participants in the experimental arm, and 5% in the control arm would continue exclusive breastfeeding for one month. Therefore 25 women would need to be enrolled in each group to detect this difference. We anticipated that 50% of participants would drop out during the trial because of the difficulties that women with inverted nipples face during breastfeeding. Hence the sample size was inflated to a total of 100 participants.

### Statistical methods

Continuous data were summarized as means (*SD*) or medians (*IQR*) as appropriate, and categorical data as counts and proportions. Because of the small sample size of the two groups, they were compared using Mann Whitney test for the continuous variables and Fisher’s Exact test for the categorical variables. Rates, relative risk (RR) and 95% confidence intervals for exclusive breastfeeding and mixed feeding at one, three, and six months were calculated using non-parametric tests. We imputed missing data on breastfeeding outcomes by using the last observation carried forward, if on last follow up the infant was reported to be on artificial milk, as it was unlikely that those infants would be shifted back to breastfeeding or mixed feeding after one month. For infants who on last follow up were on exclusive breastfeeding or on mixed feeding, this information was not imputed and was recorded as missing because those infants may have continued with the same type of nutrition or changed to mixed feeding or artificial formula later. For other missing data we used the average value for continuous variables, and random replacement to maintain proportions for categorical variables, as deemed appropriate. All analyses were conducted based on the intention to treat principle using the Statistical Package for Social Sciences version 24. A *p* value of <0.05 indicates statistical significance.

## Results

### Baseline characteristics

Between June 2018 and January 2020, 100 women meeting our inclusion criteria were approached for enrolment in the study. Of these, only 54 (54%) accepted to participate in the trial. Due to the COVID-19 pandemic, the Institutional Review Board mandated stopping of all research activities requiring face-to-face contact with participants in February 2020. Hence further recruitment to the trial was not possible and the trial ended early before reaching the intended sample size of 100 participants. Hence, we are reporting the trial as a pilot study.

**Fig. 1:**
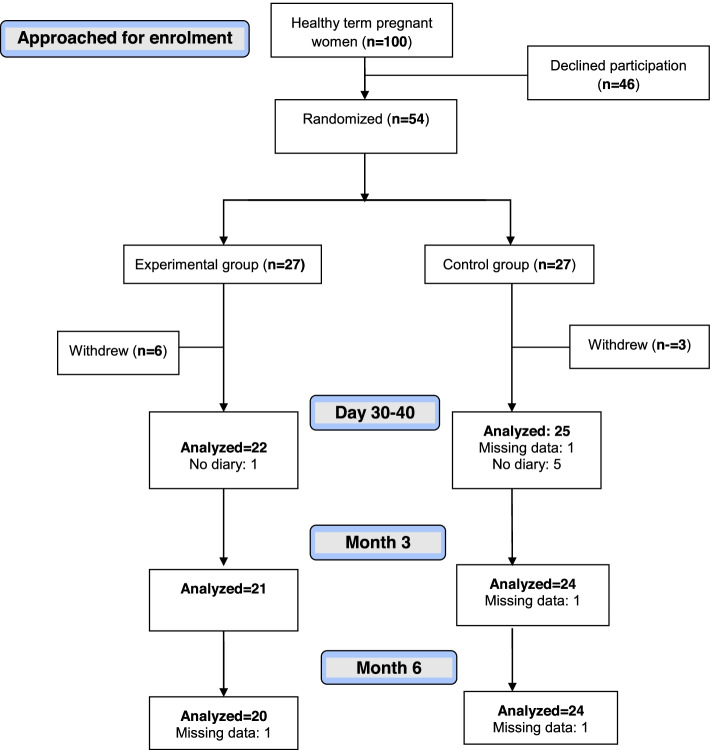
CONSORT flow diagram

The flow of the participants through the trial is summarized in the Fig. 1. In total, 9 of 54 (20%) participants withdrew, a challenge that we anticipated a priori since women with inverted nipples struggle during breastfeeding. Moreover, six participants stated that they lost their diaries. Hence, detailed information on their infants’ nutrition, breastfeeding-associated complications and other challenges encountered during the first month was unavailable. Missing data from diaries were not imputed. Hence, there were 39 participants with complete datasets: 20 in the experimental and 19 in the control group. However, we had information on breastfeeding outcomes for 47 participants at one month, 45 participants at three months, and 44 participants at six months which allowed us to conduct the planned intent to treat analysis.

Our participants had a mean (*SD*) age of 30.3 (5.0) years, and a mean (*SD*) gestational length of 38.3 (1.3) weeks. The majority 36 (66.7%) were employed, attained university education (*n*= 50; 92.6%), and had a monthly household income above $1000 (*n*=45; 83.3%). Half (*n*=27) were delivered by Cesarean section, and most reported having support at home (*n*=50; 92.6%). Of the 54 participants initially recruited, 36 (66.7%) were primi-parous and 18 (33.3%) were multi-parous mothers having a median (*IQR*) of 1.0 (1.0, 2.0) child, with a range of 1 to 4 children. The median (*IQR*) number of breastfed children among multi-parous mothers was 0.0 (0.0, 1.0) with a range of 0 to 3 children. Their median (*IQR*) longest duration of previous breastfeeding was 3.0 (1, 7.5) months. Only two (3.7%) participants reported having previous breast surgery. Flat nipples were present in 32 (59.3%) right breasts and 27 (50%) left breasts, whereas 10 (18.5%) right and 13 (24.1%) left nipples had grade 1 inversion. Grade 2 inversion was present in only 2 (3.7%) right and 5 (9.3%) left nipples. The prevalence of bilateral nipple abnormality (flatness and/or inversion) was 70.4% (*n*=38). Because flat or grade 1 inverted nipples are milder abnormalities than grade 2 inverted nipples, we merged them together in one group during analyses. There were no differences in any of the baseline characteristics of the two trial groups (Table 1).

**Table 1:** Baseline characteristics (*N*=54)

**Variable**	**Experimental** ***n*=27**	**Control** ***n*=27**	***P***
**Continuous variables** ***Median (IQR)***			
** Age (years)**	29.0 (28.0, 33.0)	29.0 (26.0, 33.0)	0.883
** Gestational length (weeks)**	38.0 (37.0, 39.0)	39.0 (38.0, 40.0)	0.231
** Number of children**	1 (1, 2)	1 (1, 2)	0.378
** Number of breastfed children**	0 (0, 1)	0 (0, 1)	0.465
** Longest previous breastfeeding (months)**^**a**^	3.0 (1.5, 10.0)	3.0 (1.0, 9.0)	0.681
** Infant birth weight (grams)**	3315 (2955, 3545)	3285 (3135, 3450)	0.647
** 1-minute APGAR score**	8 (8, 9)	9 (8, 9)	0.977
** 5-minute APGAR score**	9 (9, 10)	9 (9, 10)	0.788
**Categorical Variables *n* (%)**			
** Caesarean delivery**	16 (59.3)	11 (40.7)	0.276
** Male infant**	14 (51.9)	13 (48.1)	1.000
** Infant admitted to NICU**	1 (3.7)	1 (3.7)	1.000
** Female obstetrician**	10 (37.0)	12 (44.4)	0.782
** Mother employed**	17 (63.0)	19 (70.4)	0.773
** University education**	24 (88.9)	26 (96.3)	0.610
** Monthly income >$1000**	23 (85.2)	22 (81.5)	1.000
** Had support at home**	25 (92.6)	25 (92.6)	1.000
**Right nipple**			
** Inverted grade I**	23 (85.2)	20 (74.1)	1.000
** Inverted grade 2**	1 (3.7)	1 (3.7)	
**Left nipple**			
** Inverted grade I**	20 (74.1)	20 (74.1)	0.352
** Inverted grade 2**	1 (3.7)	4 (14.8)	
** Bilateral nipple inversion**	20 (76.9)	18 (66.7)	0.544

^a^In multiparous women

*IQR* interquartile range, *NICU* neonatal intensive care unit

### Breastfeeding outcomes

Table 2 summarizes the breastfeeding outcomes of both groups. Participants in the control group were significantly more likely to be exclusively breastfeeding at one (*RR* = 0.65, 95% *CI*: 0.44, 0.95), and at three months (*RR* = 0.66, 95% *CI*: 0.47, 0.91). They also had higher rates of any breastfeeding at six months (*RR* = 0.54, 95% *CI*: 0.34, 0.87). The two groups had similar rates of exclusive breastfeeding at six months, and of any breastfeeding at one and three months. The median (*IQR*) duration of mixed feeding was 67.5 (30.0, 120.0) days in the experimental group, and 120.0 (10.0, 180.0) days in the control group. This difference however did not achieve statistical significance (*p*=0.237). Moreover, there were no significant differences in the rates of exclusive breastfeeding or any breastfeeding between participants with bilateral and unilateral nipple abnormality at any time point (all *p* values > 0.05).

**Table 2:** Breastfeeding outcomes of trial participants

**Variable**	**Experimental** ***n/N (%)***	**Control** ***n/N (%)***	***p***	**RR (95% *CI*)**
EBF at 1 m	3/22 (13.6)	11/25 (44.0)	0.029	0.65 (0.44, 0.95)
EBF at 3 m	1/21 (4.8)	9/24 (37.5)	0.012	0.66 (0.47, 0.91)
EBF at 6 m	1/20 (5.0)	5/24 (20.8)	0.198	0.83 (0.66, 1.05)
Any BF at 1 m	18/21 (85.7)	18/24 (75.0)	0.469	1.75 (0.50, 6.15)
Any BF at 3 m	8/21 (38.1)	14/24 (58.3)	0.236	0.67 (0.38, 1.20)
Any BF at 6 m	3/20 (15.0)	13/24 (54.2)	0.011	0.54 (0.34, 0.87)

*EBF* exclusive breastfeeding, *m* month, *RR* relative risk

Of the 27 participants in the experimental group, only three (14.3%) reported using the inverted syringe technique in at least 50% of breastfeeds during the first month, and none used it afterwards. Breastfeeding by direct latching of the infant on the breast was reported by 14 (59.1%) mothers in the experimental group, and 13 (66.7%) mothers in the control group (*p* = 0.607). The nipple everted/inversion grade improved in 9 (42.5%) of the control and 6 (33.3%) of the experimental group (*p* = 0.742). By six months, more women in the control (*n*=10; 41.7%), as compared to the experimental group (*n*=5; 9.5%) were using a breast pump to express maternal milk (*p* = 0.020).

Breastfeeding-associated complications were reported by a similar number of participants at one month: 9 (52.9%) in the experimental group versus 8 (42.1%) in the control group, *p* = 0.739. However, at three months more mothers in the control group (*n*=9; 37.5%) as compared to the experimental group (*n*=1; 4.8%) had one or more breastfeeding-associated complication (*p*=0.012).

### Maternal breastfeeding satisfaction and quality of life

The two groups had similar scores on the Arabic Maternal Breastfeeding Evaluation Scale and its three subscales: Infant Satisfaction/Growth, Maternal Enjoyment/Role Attainment, and Lifestyle/Body Image. They also had comparable scores on the Maternal Postpartum Quality of Life Questionnaire and its five domains: Health functioning, Relational/family-friends, Socioeconomic, Relational/husband-partner, and Psychological/Baby (Table 3).

**Table 3:** Scores of participants on Maternal Postpartum Quality of life Questionnaire and Maternal Breastfeeding Evaluation Scale (*N*=49)

**Variable**	**Experimental** ***n*=22** *Median (IQR)*	**Control** ***n*=24** *Median (IQR)*	***p***
**Maternal Postpartum Quality of Life**			
Overall score	23.5 (21.4, 25.6)	23.1 (20.6, 25.2)	0.495
Health functioning	22.4 (20.3, 24.4)	20.9 (17.9, 24.7)	0.166
Relational/family-friends	22.6 (19.7, 23.9)	20.7 (16.8, 22.7)	0.113
Socioeconomic	24.2 (22.3, 26.8)	25.9 (23.7, 27.9)	0.422
Relational/husband-partner	27.6 (25.0, 30.0)	29.4 (25.2, 30.0)	0.486
Psychological/Baby	23.2 (20.5, 26.7)	23.8 (17.0, 26.3)	0.852
**Arabic Maternal Breastfeeding Evaluation Scale**	***n*=23**	***n*=26**	***p***
Overall score	103.0 (99.0, 111.0)	105.5 (92.8, 112.3)	0.920
Infant Satisfaction/Growth	37.0 (34.0, 39.0)	38.0 (34.8, 41.0)	0.393
Maternal Enjoyment/Role Attainment	49.0 (46.0, 51.0)	48.5 (44.8, 53.0)	0.856
Lifestyle/Body Image	20.0 (16.0, 21.0)	18.0 (14.8, 21.3)	0.231

*IQR* interquartile range

## Discussion

In this study, we found that the use of the inverted syringe technique in women with inverted nipples was not associated with improvement in breastfeeding rates at any time point during the first six months post-partum, nor was it associated with higher maternal satisfaction with breastfeeding, or better quality of life. Moreover, the fact that few participants in the experimental group used the inverted syringe suggests that they may have found it to be cumbersome or impractical to do with every breastfeeding. It is interesting to note that breastfeeding rates and breastfeeding-associated complications were higher in the control group. These findings may be due to more women in the control group using breast pumps, as compared to participants in the experimental group.

Our study suffers from a major limitation related to its small sample size. To start with, recruitment into the trial was challenging. About 50% of eligible women who were approached for enrolment declined, which may suggest lack of acceptance of the intervention, negative previous breastfeeding experiences that discouraged them from experimenting with a new intervention, or lack of intent to breastfeed. A second contributor to the small sample size was the high attrition rate which was anticipated a priori. Finally, the premature stopping of the trial mandated by the IRB during the COVID-19 pandemic was the major barrier to recruiting the desired sample size. The trial therefore is underpowered to detect a difference between the two groups, should a difference exist. The differences we found in the rates of breastfeeding and breastfeeding-associated complications should be interpreted with caution, as they may be due to chance. Due to the small sample size, we did not conduct a multivariate logistic regression analysis as planned in our protocol to examine the association between the breastfeeding outcome and different predictors.

The strength of this study is that it is the only randomized clinical trial investigating the effectiveness of the inverted syringe technique. All previous reports about the use of non-surgical techniques (including the inverted syringe) in women with inverted nipples were case series [17–19]. Hence the reported success rates in these studies may have been affected by inherent selection and/or confounding biases.

Despite its limitations, we believe that it is important to report our findings as the study may be a stepping stone for further research on the effectiveness of non-surgical treatments of inverted nipples in lactating mothers. It sheds light on the need for larger clinical trials in different settings, since acceptance of the intervention may be different in other populations/settings. Qualitative research is also needed to explore the lack of acceptance of this technique in our context.

## Supplementary Information

**Additional file 1. **Anonymized dataset.

## Data Availability

The dataset supporting the conclusions of this article is included within the article and its additional file.

## Abbreviations

SD: Standard deviation
IQR: Interquartile range
RR: Relative risk
CI: Confidence interval
COVID-19: Coronavirus of 2019
